# Supplementary material for: Genetic predisposition to elevated BMI and adult asthma phenotypes in a Japanese population
Source: PLoS One. 2026 Jan 29;21(1):e0340728. doi: 10.1371/journal.pone.0340728 (PMC12854457; doi:10.1371/journal.pone.0340728)
Supplement: Attachment — Submitted filename: Review report.docx [file pone.0340728.s002.docx]

This is a comprehensive and interesting study about the association between adult onset asthma and BMI, in a large cohort of subjects. A minör point to critise is the mean BMI of patients and healthy controls is not matched! It is better to analyse at least a subgroup of controls with a matched BMI with the asthmatics.
