# Supplementary material for: Genetic predisposition to elevated BMI and adult asthma phenotypes in a Japanese population
Source: PLoS One. 2026 Jan 29;21(1):e0340728. doi: 10.1371/journal.pone.0340728 (PMC12854457; doi:10.1371/journal.pone.0340728)
Supplement: Attachment — Submitted filename: Response to Reviewers_0926.docx [file pone.0340728.s004.docx]

**Review Comments to the Author**

**Reviewer #1**

**Comment #1:** Line 35 isn't justified based on the finding - suggest remove 'asthma may contribute to increased BMI.'

**Response #1:** Based on the comments, we have revised line 35 of the abstract. The revised version reads: “This study demonstrates a genetic heterogeneity in the phenotype of adult asthma among a Japanese population, showing that genetic variants associated with BMI contribute to specific subtypes of asthma.”

In addition, the following revisions were made to adjust the future research direction and ensure consistency throughout the abstract, focusing on a longitudinal approach to understand temporal relationships rather than assuming bidirectional causality.

“Prospective longitudinal studies are essential to delineate the interactions between genetic predisposition, elevated BMI, subsequent changes in adiposity, and the evolution of asthma phenotypes, which would facilitate the development of mechanism-based therapeutic strategies tailored to genetically-defined patient subgroups.”

**Comment #2:** Comparison should have been made between asthma vs overweight asthma and overweight vs overweight asthma.

**Response #2:** In the revised manuscript, new Figure 5 shows a comparison of BMI-GRS between four groups: non-obese controls, obese controls, non-obese asthmatics and obese asthmatics. The obese control group had the highest BMI-GRS, while the non-obese control group had the lowest. No significant difference in BMI-GRS was noted between non-obese asthma and obese asthma (corrected *p*= 0.56).

**Comment #3:** Why 2 different cohorts?

**Response #3:** Our sequential recruitment strategy (Tsukuba Cohort 2: 2008–2012; Tsukuba Cohort 1: 2013–2022) was designed to maximize the number of participants and thereby maximize the statistical power and scientific rigor while addressing practical and ethical considerations (PLoS One 2024;19(3):e0300000. doi:10.1371/journal.pone.0300000.). The decade-long recruitment period and consistent geographic focus on Ibaraki Prefecture enhance representativeness while maintaining genetic homogeneity within the study’s Japanese population. However, recruiting over 2,000 participants with comprehensive clinical and genetic data within a short time frame presents significant ethical and logistical challenges. Our phased approach enabled continuous patient enrollment from Tsukuba University Hospital and its affiliated institutions.

**Comment #4:** Why were different SNP genotyping methods used?

**Response #4:** The evolution of genotyping technology during our extended collection period necessitated platform updates. For Tsukuba Cohort 2, Illumina HumanHap 550K/610-Quad BeadChips provided comprehensive, genome-wide coverage, representing the gold standard from 2008 to 2012. For Tsukuba Cohort 1, the Infinium Asian Screening Array-24 v1.0 was optimized specifically for East Asian populations and incorporated population-specific variants with enhanced coverage of Asian-specific alleles.

**Comment #5:** Incomplete summary for rs939584 in table.

**Response #5:** We have corrected the summary for rs939584 in Table 1.

**Comment #6:** Duplication of SNP selection and BMI-GRS score in methods and results.

**Response #6:** Thank you for identifying this methodological redundancy. We have addressed this duplication by removing the detailed description of SNP selection and BMI-GRS calculation from the Results section while retaining the comprehensive methodological details in the Methods section.

**Comment #7:** Line 206-212 should be in discussion

**Response #7:** According to the comment, we have moved these sentences to discussion Line 305 to 308 in the revised manuscript.

**Comment #8:** Table 2 - Mann Whitney spelling error. Was there a weighted difference in BMI due to the difference in number of healthy and asthmatic participants for a difference of 0.52 to be significant?

**Response #8:** The spelling mistake the reviewer pointed out has been corrected.

We believe that the significance is not due to an artefact of "weighted difference" from unequal sample sizes. The large, combined sample size (n = 2,267) increased statistical power and reduced the standard error, enabling the precise detection of a small but genuine difference. While this 0.52 BMI difference is statistically significant, its clinical significance should be evaluated separately from the statistical result.

**Comment #9:** A graph showing no correlation between BMIGRS and asthma should be included.

**Response #9:** Thank you for this suggestion. Figure 4B shows no correlation between BMI-GRS and asthma. We confirmed that asthma is not associated with the BMI-GRS, even when the analysis was adjusted for BMI (p = 0.87).

**Reviewer #2:**

**Comment #1:** This is a comprehensive and interesting study about the association between adult onset asthma and BMI, in a large cohort of subjects. A minör point to critise is the mean BMI of patients and healthy controls is not matched! It is better to analyse at least a subgroup of controls with a matched BMI with the asthmatics.

**Response #1:** We appreciate your thoughtful suggestion regarding BMI matching. However, we avoided this approach in our study design for several critical methodological reasons. First, our BMI-GRS formula was constructed using the entire healthy cohort (n = 1,532) to maximize statistical power and generalizability to the Japanese population. Of the 85 previously identified BMI-associated SNPs, only six showed significant associations in our cohort. Restricting the discovery sample further through BMI matching would substantially increase the risk of false negatives and compromise the validity of the genetic risk score. Second, the two-phase approach (discovery in healthy individuals and application to disease cohorts) is the gold standard in genetic epidemiology. This approach ensures that genetic risk scores reflect population-level genetic architecture rather than disease-specific patterns. Restricting the BMI range in our discovery cohort would introduce systematic bias in genetic effect estimation and reduce the generalizability of our findings to the broader Japanese population.

However, to address the reviewer’s concern without compromising our BMI-GRS construction, we conducted BMI-adjusted models to estimate the direct effects of the BMI-GRS by comparing non-asthmatic healthy controls with patients with asthma. This analysis confirmed that asthma is not associated with the BMI-GRS, even when adjusted for BMI (p = 0.87). We have mentioned the result in the revised manuscript (Line 220 to 222).

**Reviewer #3:** The study aimed to investigate the role of genetic predisposition to increased BMI, evaluated by calculating a BMI-genetic risk score (BMI-GRS), on adult asthma phenotypes and explore whether these genetic factors might distinguish certain subgroups of overweight or obese asthma patients. The study included 1530 healthy individuals and 735 adult asthma patients. Six single nucleotide polymorphisms (SNPs) which were confirmed as having an association with BMI in the healthy population in the study were used to calculate BMI-GRS. The authors found that patients with asthma had significantly higher BMI values compared to non-asthmatic healthy controls, but there was no significant difference in BMI-GRS between the two groups. The study reported that no significant correlation was found between BMI and BMI-genetic risk scores in asthmatic patients. The authors performed a cluster analysis using age of asthma onset, %FEV1, BMI and BMI-GRS. This analysis identified 6 distinct asthma patient groups, among these, two clusters were characterized by increased BMI, one with a higher BMI-GRS and the other with a lower BMI-GRS. I have some major and minor comments.

**Comment #1:** Lines 174-178, the population attributable risk fraction (PARF) was calculated assuming there are no confounding factors, and no confounding adjustment in the calculation of PARF was performed. Additionally, PARF assumes that a cause-effect relationship exists between exposure and outcome. However, the study reported that there was no significant correlation between BMI and BMI-genetic risk scores in asthmatic patients. This is a limitation.

**Response #1:** Thank you for raising these important methodological concerns about our PARF analysis. After careful consideration of the reviewer feedback and our study's key findings, we have decided to remove the PARF analysis entirely from the manuscript. The lack of correlation between BMI-GRS and BMI in asthmatic patients violates the basic causal assumptions required for valid PARF interpretation. In fact, the presence of high BMI-GRS cluster among non-obese asthmatic patients fundamentally challenges the traditional mediation pathway (BMI-GRS → BMI → obesity-related asthma) that underlies PARF calculations. We now consider this finding indicates that BMI-associated genetic variants influence asthma through multiple biological pathways, including both BMI-associated and BMI-independent mechanisms.

In the revised manuscript, we have replaced PARF with a multinomial logistic regression model (7 categories including healthy controls) to quantify genetic risk profiles across asthma phenotypes that provide a more accurate and comprehensive assessment of genetic heterogeneity in asthma pathogenesis while avoiding the methodological limitations inherent in PARF calculations under our study conditions.

**Comment #2:** - Abbreviations should be added to Table 1. In table 1 the summary part of rs939584 is not completed.

**Response #2:** We have added the relevant abbreviations as a footnote to Table 1. We have also corrected the summary for *rs939584* in Table 1.

**Comment #3:** - Lines 187-199 of the results section is about methods.

**Response #3:** Thank you for identifying this methodological redundancy. We have addressed this duplication by removing the detailed description of SNP selection and BMI-GRS calculation from the Results section while retaining the comprehensive methodological details in the Methods section.

**Comment #4:** - Line 197, table 1a, 1b, there is no table 1a or 1b.

**Response #4:** As you correctly noted, there is only Table 1, and it was mistakenly referred to as Table 1A and 1B. We have corrected the text so that it consistently refers to Table 1.

**Comment #5:** - Lines 206-212 of results section would be more appropriate in discussion.

**Response #5:** According to the comment, we have moved these sentences to discussion (line 305 to 308) in the revised manuscript.

**Comment #6:** - I suggest adding more clinical characteristics on table 2, like BMI groups, obesity and overweight rates.

**Response #6:** In accordance with your comment, we have added additional clinical characteristics to Table 2, including the number and percentage of participants with BMI ≥25 and BMI ≥30 in both the healthy control and asthma groups.

**Comment #7:** - I suggest adding the criteria defining atopy to the methods section.

**Response #7:** The definition of atopy has been added to the Study participants section of Material and Methods.

**Comment #8:** - Lines 229-235 of the results section is about methods.

**Response #8:** We have moved the sentences to the methods section in the revised manuscript.

**Comment #9:** - The authors stated in lines 268-270 that ‘Our results suggest that genetic factors influencing BMI may play a crucial role in the development of adult-onset overweight/obese asthma in certain patients, particularly those in Cluster-5, where a higher BMI-genetic risk score (BMI-GRS) was observed.’. For this comment multivariate analyses should be performed. Additionally, no significant correlation was found between BMI and BMI-genetic risk scores in asthmatic patients in the study.

**Response #9:** We sincerely appreciate this crucial methodological critique, which has prompted us to fundamentally reconsider our analytical approach and transform our interpretation of key findings. Rather than viewing the lack of BMI-GRS/BMI correlation in asthmatic patients as problematic, we now recognize this as a pivotal discovery providing direct evidence for genetic heterogeneity and pathway diversity in asthma pathogenesi*s.*

We conducted multivariable logistic regression modeling comparing BMI-GRS contribution to each cluster adjusting for age, sex, smoking history and BMI, explicitly framed as descriptive evidence for genetic heterogeneity rather than predictive modeling. Among the six distinct asthma phenotypes identified, we found two overweight/obese clusters (one with elevated BMI-GRS, one without) and four non-obese clusters (with one showing significantly elevated BMI-GRS). This pattern demonstrates that BMI-associated genetic variants contribute to asthma susceptibility through heterogeneous biological mechanisms spanning the entire BMI spectrum - both BMI-mediated pathways (traditional obesity-related mechanisms) and BMI-independent pleiotropic pathways.

We have reframed our conclusions from causal prediction to evidence-based genetic heterogeneity assessment. "BMI-GRS plays a crucial role in development of Cluster-5 asthma" was revised to read " BMI-associated genetic variants showed differential distribution patterns across six distinct asthma clusters, with elevated BMI-GRS observed in both obese and non-obese phenotypes. These descriptive findings suggest that BMI-associated genetic variants may influence asthma susceptibility through multiple biological pathways. "

**Comment #10:** - Lines 276-285, If clinical features are not different among the two phenotypes (cluster 5 and 6), what is the added value of BMI-GRS calculation? I suggest discussing this.

**Response #10:** Thank you for this suggestion. We believe that the added value of BMI-GRS lies not in identifying current different phenotypes, but in revealing unique pathogenic mechanisms that may be useful for prognosis, treatment selection, and risk stratification. This represents a paradigm shift from reactive phenotype-based medicine to predictive, genetically informed precision medicine, and has the potential to transform the current single ‘obese asthma’ category into a genetically stratified approach, even when clinical findings are similar, thereby having a concrete impact on personalized patient care. This issue is discussed in the revised manuscript.

**Comment #11:** - I suggest adding clinical comparison of all 6 clusters to the results section.

**Response #11:** Thank you. Based on your suggestion, we have added clinical comparison of all 6 clusters to the result section as follows: We identified six clusters with distinct clinical profiles. Cluster 1 was characterized by early-onset asthma with the longest disease duration and a lean body habitus; despite their prolonged disease course, these patients maintained relatively preserved lung function. Cluster 2 represented elderly patients with a substantial smoking history who exhibited the most severe airflow limitation, while their BMI and genetic risk for obesity remained moderate. Cluster 3 also included elderly patients but was distinguished by the lowest BMI-GRS, indicating minimal genetic predisposition to obesity; these individuals were relatively lean and preserved good lung function. In contrast, Cluster 4 comprised patients with the latest asthma onset and higher BMI-GRS, reflecting a strong genetic tendency toward obesity, yet they maintained preserved pulmonary function. Cluster 5 was defined by obesity combined with the highest BMI-GRS, representing individuals with both a phenotypic and genetic predisposition to obesity and showing moderate airflow limitation. Finally, Cluster 6 also displayed an obese phenotype but was associated with relatively low BMI-GRS, suggesting that non-genetic factors may predominantly underlie obesity in this group; their lung function was moderately impaired.

**Comment #12:** - In lines 294-296, it is stated that ‘Specifically, asthma symptoms may lead to weight gain in some patients due to reduced physical activity or medication side effects, while genetic predispositions to obesity may increase the risk of developing asthma in others.’. I suggest adding previous literature data and a detailed discussion.

**Response#12:** Thank you for your proposal. The association between asthma and obesity is increasingly recognized as a bidirectional and mechanistically complex relationship [1-3]. Our genetic findings must be interpreted within this established framework of multiple, interconnected pathways that operate simultaneously across different patient subgroups.

To thoroughly verify the established pathway, we have significantly expanded this section based on extensive literature evidence, as described below (Line 309 to 324).

Obesity-to-asthma pathways include mechanical effects (reduced chest wall compliance and lung volumes leading to airway closure and hyperresponsiveness [4,5]), systemic inflammation (elevated TNF-α/IL-6 and altered adipokines with increased ratio of leptin/adiponectin promoting Th1/Th17 and neutrophilic inflammation [1,6]), metabolic dysregulation (increased ADMA reducing nitric oxide bioavailability [8]), hormonal influences (dysregulated sex hormones affecting airway inflammation [7]), and gut-lung axis changes (obesity-related dysbiosis sustaining systemic inflammation [2]). Asthma-to-obesity pathways include reduced physical activity due to exertional dyspnea or fear of exacerbation [1,4], and chronic corticosteroid-induced weight gain through metabolic effects including increased appetite and insulin resistance [10]. Our study reveals critical genetic heterogeneity within obesity-related asthma, identifying two etiologically distinct phenotypes despite similar BMI levels—Cluster-5 with significantly elevated BMI-GRS and Cluster-6 with significantly reduced BMI-GRS. Cluster-5 represents genetically driven obese asthma, while Cluster-6 suggests environmentally driven obese asthma, where obesity results primarily from asthma-to-obesity pathways. This genetic stratification within phenotypically similar obese asthmatics provides novel insights into asthma heterogeneity and supports precision medicine approaches.

References:

[1] Tooba R, Wu TD. Respir Med. 2022;204:107012.

[2] Tashiro H, et al. Allergol Int. 2024.

[3] Olejnik AE, et al. J Clin Med. 2024;13:3474.

[4] Peters U, et al. Expert Rev Respir Med. 2018;12(9):755-767.

[5] Salome CM, et al. J Appl Physiol. 2010;108(1):206-11.

[6] Peters MC, et al. Lancet Respir Med. 2016;4(7):574-84.

[7] Zein JG, Erzurum SC. Proc Am Thorac Soc. 2015;12(1):36-45.

[8] Holguin F, et al. Am J Respir Crit Care Med. 2013;187(2):153-9.

[9] Holguin F, et al. J Allergy Clin Immunol. 2011;127(6):1486-93.e2.

[10] Fardet L, et al. J Clin Endocrinol Metab. 2021.]

This expansion demonstrates how our genetic stratification findings contribute novel insights to the established bidirectional obesity-asthma framework.

**Comment #13:** - In lines 297-299, it is stated that ‘In conclusion, this study supports the growing body of evidence suggesting that genetic susceptibility to higher BMI is a key factor in the development of adult-onset asthma in certain subgroups of overweight or obese patients.’ As the study does not include a multifactorial analysis and a longitudinal analysis, I suggest to revise the conclusion part to be supported by the data.

**Response #13:** Based on the results of the newly conducted multinomial logistic analysis, we have reframed our conclusions from causal prediction to evidence-based genetic heterogeneity assessment (Line 344 to 352). “this cross-sectional study demonstrates genetic heterogeneity within adult asthma phenotypes in a Japanese population. BMI-associated genetic variants showed differential distribution patterns across six distinct asthma clusters, with elevated BMI-GRS observed in both obese and non-obese phenotypes. These descriptive findings suggest that BMI-associated genetic variants may influence asthma susceptibility through multiple biological pathways. This represents a paradigm shift from reactive phenotype-based medicine to predictive, genetically informed precision medicine, and has the potential to transform the current single ‘obese asthma’ category into a genetically stratified approach, even when clinical findings are similar.”

**Comment #14:** - The discussion section of the manuscript includes only one reference. The discussion section needs a revision and expansion to discuss the results with other previous studies.

**Response #14:** In light of your comments and those of other reviewers, we have reinterpreted the research results and made significant revisions to both the discussion section and the abstract and expanded the number of references to previous studies as suggested by the reviewer.

**Commet #15:** - I suggest giving percentages in the x axes of figure 4.

**Response #15:** Thank you for this suggestion. In response, we have created a new bar chart in which the x-axis is expressed in percentages, in addition to the original Figure 4.

**Reviewer #4:** I read with interest the manuscript entitled „Impact of hereditary predisposition to increased BMI on adult asthma phenotype”. The manuscript describes the results of a study delving into the genetic susceptibility to elevated body mass index (BMI) and its subsequent influence on adult-onset asthma, specifically within a Japanese cohort. This study indicates a bidirectional relationship: genetic predisposition to a higher BMI appears to contribute to adult-onset overweight/obese asthma, and conversely, asthma itself may lead to increased BMI. Therefore, the study provides valuable results that contribute to progress in the fields of precision medicine and precision nutrition.

The manuscript is generally well-written. Nevertheless, some aspects of the manuscript should be addressed before a decision on publication. The manuscript needs revision focused on the remarks stated below.

MAJOR REMARKS:

**Comment #1:** Title: Please consider reformulating the title so that it is understood that the study was conducted in Japan. This suggestion is made in the context that the Japanese population has particularities regarding being classified as overweight/obese in relation to BMI.

**Response #1:** Thank you. According to your suggestion, we have revised the title to “Genetic predisposition to elevated BMI and adult asthma phenotypes in a Japanese population” to emphasize the Japanese population context.

**Comment #2:** Line 42: Please provide recent data regarding the prevalence of both obesity and asthma, at global level and in Japan, respectively.

**Response #2:** We have revised Line 42 to include recent global and Japan-specific prevalence data, which we believe significantly strengthens the public health rationale and contemporary relevance of our research. "Obesity and asthma represent major global health challenges, with more than 1 billion people worldwide living with obesity and asthma affecting approximately 262 million people globally [WHO, 2022; GBD 2019]. In Japan, 33.0% of men and 22.3% of women exceed the Asian clinical threshold for overweight/obesity (BMI ≥25 kg/m²), while adult asthma prevalence is approximately 8-10% [Ministry of Health, Labour and Welfare, 2020]. The intersection of these conditions is of particular clinical importance, as overweight and obese individuals have 1.17-1.62 and 1.92 higher odds of developing asthma, respectively, compared to those with normal BMI [*Beuther DA, Sutherland ER. Overweight, obesity, and incident asthma: a meta-analysis of prospective epidemiologic studies. Am J Respir Crit Care Med. 2007;175(7):661-666. doi: 10.1164/rccm.200611-1717OC*]."

References to be added:

1. World Health Organization. Global Health Observatory: Obesity and overweight. Geneva: WHO; 2022
2. GBD 2019 Diseases and Injuries Collaborators. Global burden of 369 diseases and injuries in 204 countries and territories, 1990-2019. Lancet. 2020;396(10258):1204-1222
3. Ministry of Health, Labour and Welfare, Japan. National Health and Nutrition Survey 2019. Tokyo: MHLW; 2020
4. Ministry of Health, Labour and Welfare, Japan. Patient Survey 2020. Tokyo: MHLW; 2021

**Comment #3:** Line 45: Please define BMI.

Response #3: In accordance with your suggestion, we have provided a definition of BMI in the study participants section of Material and Methods. Specifically, BMI is defined as weight in kilograms divided by the square of height in meters (kg/m²).

**Comment #4:** Lines 45-49: **The mechanisms by which obesity contributes to the pathogenesis of asthma need to be described more extensively,** citing a significantly larger number of scientific publications. For instance, potential mediators between obesity and asthma should be briefly described (e.g., mechanical changes, systemic inflammation, sex hormones, arginine metabolism, gut microbiome) (https://pmc.ncbi.nlm.nih.gov/articles/PMC9671155/#S3; https://www.sciencedirect.com/science/article/pii/S1323893023000837?via%3Dihub#sec3). Asthma phenotypes in obesity (e.g., Late-Onset Asthma Phenotype, Early-Onset Asthma

Phenotype) should also be mentioned (<https://pmc.ncbi.nlm.nih.gov/articles/> PMC11204497/#sec5-jcm-13-03474).

**Response #4:** Thank you for this suggestion. We have significantly revised the Introduction to incorporate the suggested mechanisms and phenotypes with appropriate citations as described below.

"Obesity increases the risk of asthma through multiple well-characterized pathways [1,2]. Mechanical effects include reduced chest wall compliance and diaphragmatic excursion, leading to diminished lung volumes, airway dysanapsis, and hyperresponsiveness [4,5]. Systemic inflammation plays a central role, as adipose tissue secretes pro-inflammatory cytokines (TNF-α, IL-6) and altered adipokines (elevated leptin, reduced adiponectin) that promote Th1/Th17 responses and neutrophilic airway inflammation [1,6]. Metabolic pathways contribute through elevated asymmetric dimethylarginine (ADMA), which reduces nitric oxide bioavailability and impairs bronchodilation [8]. Hormonal influences, particularly dysregulated sex hormones in obese women, modulate airway inflammation and responsiveness [7]. Additionally, the gut-lung axis operates through obesity-related dysbiosis, altering microbial metabolites and increasing endotoxemia [2]. These mechanisms give rise to distinct obesity-associated asthma phenotypes, including late-onset, non-atopic, severe asthma in obese adults and early-onset allergic asthma that worsens with subsequent weight gain [3,9]"

References to be added:

[1] Tooba R, Wu TD. Obesity and Asthma: A Focused Review. Respir Med. 2022;204:107012.

[2] Tashiro H, Kurihara Y, Kuwahara Y, Takahashi K. Impact of obesity in asthma: Possible future therapies. Allergol Int. 2024 Jan;73(1):48-57. doi:10.1016/j.alit.2023.08.007. Epub 2023 Sep 1. PMID: 37659887.

[3] Olejnik AE, Larkin EK, Bleecker ER, et al. Association of Obesity and Severe Asthma in Adults. J Clin Med. 2024;13:3474.

[4] Peters U, Dixon AE, Forno E. The effect of obesity on lung function. Expert Rev Respir Med. 2018;12(9):755-767.

[5] Salome CM, King GG, Berend N. Physiology of obesity and effects on lung function. J Appl Physiol. 2010;108(1):206-11.

[6] Peters MC, McGrath KW, Hawkins GA, et al. Plasma interleukin-6 concentrations, metabolic dysfunction, and asthma severity. Lancet Respir Med. 2016;4(7):574-84.

[7] Zein JG, Erzurum SC. Asthma is different in women. Proc Am Thorac Soc. 2015;12(1):36-45.

[8] Holguin F, Comhair SA, Hazen SL, et al. An association between L-arginine/asymmetric dimethyl arginine balance, obesity, and the age of asthma onset phenotype. Am J Respir Crit Care Med. 2013;187(2):153-9.

[9] Holguin F, Baffi CW, Pohunek P, et al. Obesity and asthma: an association modified by age of asthma onset. J Allergy Clin Immunol. 2011;127(6):1486-93.e2.

**Comment #5:** Discussion section: this section must be thoroughly revised. Discussions must be based on scientific articles already published on the topic of the study. Currently, only one scientific article is cited in the discussion section, and this aspect needs to be significantly improved.

**Response #5:** Thank you. In light of your comments and those of other reviewers, we have reinterpreted the research results and made significant revisions to both the discussion section and the abstract and expanded the number of references.

.

**Comment #6:** Line 92: Please provide the date of issue of the ethical approval number.

**Response #6:** Thank you. The study was first approved by the Institutional Review Board on February 21, 2018, and the most recent approval of the amendment was obtained on March 28, 2025.

**Comment #7:** Table 1: The references in the table should be moved to the reference list and kept in the table only as numbers.

**Response #7:** In accordance with your suggestion, we have revised Table 1 by transferring the references to the reference list and leaving only their corresponding numbers in Table1.

**Comment #8:** Figure 5: Please remove the text “Figure 1” from this figure.

**Response #8:** Following your instruction, we have deleted the text “Figure 1” from the figure.
